# Microbial bioactive complex supplementation is associated with gut microbial fermentation and feed conversion in weaned piglets

**DOI:** 10.3389/fvets.2026.1863429

**Published:** 2026-07-09

**Authors:** Mi Ae Park, Soyeon Park, Hee Seop Yu, Da Jung Lim, Seoyun Son, Yong Hee Yoon, Dae-Hyuk Kim, Yangseon Kim

**Affiliations:** 1 Department of Research and Development, Center for Industrialization of Agricultural and Livestock Microorganisms, Jeongeup, Republic of Korea; 2 Jungnongbio, Jeongeup, Republic of Korea; 3 Department of Molecular Biology, Institute for Molecular Biology and Genetics, Jeonbuk National University, Jeonju, Republic of Korea; 4 Department of Bioactive Material Science, Institute for Molecular Biology and Genetics, Jeonbuk National University, Jeonju, Republic of Korea

**Keywords:** feed conversion ratio, feed efficiency, gut microbiota, microbial bioactive complex, propionate, weaned piglets

## Abstract

Improving feed efficiency while maintaining gut stability is a key nutritional objective during the post-weaning period in piglets. This study investigated the effects of dietary supplementation with a microbial bioactive complex (MBC), composed of *Pediococcus pentosaceus* CACC616, *Lactobacillus reuteri* CACC607, *L. dextrinicus* CACC889, *L. pentosus* CACC891, and *Saccharomyces cerevisiae* CACC699, on feed efficiency and gut microbial fermentation in weaned piglets. The complex was produced via solid-state fermentation (SSF), enabling the co-delivery of viable microorganisms and fermentation-derived metabolites. A total of 34 piglets were randomly assigned to either a basal diet (control) or the same diet supplemented with MBC for 33 days. The MBC contained approximately 2.4 × 10^9^ CFU/g of lactic acid bacteria and 5.17 × 10^6^ CFU/g of yeast and was included in the diet at 0.5%. MBC supplementation significantly improved feed conversion ratio (*p* < 0.05) and reduced average daily feed intake, indicating enhanced feed utilization efficiency without adverse effects on health-related blood parameters. The constituent strains exhibited desirable probiotic and safety characteristics, including tolerance to acidic and bile salt conditions, adhesion to porcine intestinal epithelial cells (IPEC-J2), absence of hemolytic activity, and low biogenic amine production. Microbial analysis revealed that taxa enriched in the MBC-treated group, including *Succinivibrio* and *Succinivibrionaceae UCG-001*, are associated with carbohydrate fermentation and succinate-producing pathways. Collectively, these findings indicate that dietary supplementation with MBC is associated with improved feed conversion and changes in fermentation-related gut microbial taxa in weaned piglets, supporting its potential application as a functional feed additive in swine production.

## 1 Introduction

Weaning represents a critical transition period in pig production, characterized by abrupt changes in diet, environment, and social conditions that often impair feed intake, nutrient utilization, and growth performance. These stressors are frequently associated with intestinal dysbiosis, disruption of barrier function, and increased susceptibility to enteric disorders, ultimately leading to reduced feed efficiency and economic losses in swine production (1, 2). Historically, in-feed antibiotics have been widely used to mitigate these challenges and improve growth performance; however, growing concerns regarding antimicrobial resistance and drug residues have led to restrictions on antibiotic growth promoters, necessitating the development of effective nutritional alternatives (3, 4).

Microbial-based feed additives have emerged as promising strategies to stabilize the gut ecosystem and enhance nutrient utilization. In addition to conventional probiotics consisting solely of viable microorganisms, increasing attention has been directed toward microbial bioactive complexes that integrate live microbial cells with fermentation-derived metabolites, enzymes, and other bioactive compounds. These complexes may exert synergistic effects on gut microbial ecology and metabolic activity, thereby influencing gut microbial fermentation processes and host performance (5–7). In particular, production via solid-state fermentation (SSF) enables the co-delivery of viable microorganisms and bioactive metabolites within a functional matrix, potentially enhancing their stability and efficacy.

Gut microbial fermentation plays a critical role in nutrient recovery and metabolic output in swine. Gut microbial fermentation in the large intestine generates short-chain fatty acids (SCFAs), which contribute to host energy metabolism and intestinal health. The balance of fermentation pathways is influenced by substrate availability and microbial composition, and shifts in gut microbial fermentation patterns may impact nutrient utilization efficiency and gut function (8, 9). Therefore, nutritional strategies that modulate gut microbial fermentation toward more favorable metabolic pathways may improve feed efficiency.

Multi-strain microbial systems may further enhance these effects through ecological complementarity and functional diversity. Different microbial species can occupy distinct ecological niches within the gastrointestinal tract, potentially improving colonization stability and metabolic interactions (10–12). However, the effects of multi-strain microbial bioactive complexes on feed efficiency and gut microbial fermentation in weaned piglets remain insufficiently characterized.

Therefore, the present study aimed to evaluate the effects of dietary supplementation with a multi-strain microbial bioactive complex composed of *P. pentosaceus*, *L. reuteri*, *L. dextrinicus*, *L. pentosus*, and *S. cerevisiae* on feed efficiency, gut microbial composition, and gut microbial fermentation in weaned piglets. The complex was produced using a solid-state fermentation process to enable the co-delivery of viable microorganisms and fermentation-derived metabolites. We proposed that dietary MBC supplementation would improve feed efficiency and modulate gut microbial fermentation during the post-weaning period.

## 2 Materials and methods

### 2.1 Acid and bile salt tolerance assay

Tolerance to artificial gastrointestinal conditions was evaluated as described by Park et al. (13). *Lactobacillus rhamnosus* GG (ATCC 53103) was used as a reference probiotic strain. Acid tolerance was assessed by suspending the cultures in sterile phosphate-buffered saline (PBS) adjusted to pH 2.5, with PBS at pH 7.2 used as a control. The suspensions were incubated at 37 °C for 2 h. For bile salt tolerance, cultures were inoculated into MRS broth or YPD broth for bacterial and yeast strains, respectively, both supplemented with 0.3% (w/v) oxgall, and incubated at 37 °C for 8 h. After incubation, samples were serially diluted (10-fold), and appropriate dilutions were plated on LAB Petrifilm and RYM Petrifilm for bacterial and yeast strains, respectively (3 M, USA). The Petrifilms were incubated at 37 °C for 24 h, and bacterial survival was determined by counting colony-forming units (CFU).

### 2.2 Cell adhesion assay

The adhesion ability of the strains to porcine intestinal epithelial cells (IPEC-J2) was evaluated with modifications to a previously described method (14). IPEC-J2 cells were cultured in Dulbecco’s Modified Eagle’s medium (DMEM, Gibco, USA) supplemented with 10% heat-inactivated fetal bovine serum (FBS, HyClone, USA) and 1% penicillin–streptomycin (Gibco, USA). Cells were maintained at 37 °C in a humidified atmosphere containing 5% CO₂. For the adhesion assay, IPEC-J2 cells were seeded in 24-well plates (SPL Life Sciences, South Korea) at a density of 1.5 × 10^4^ cells/well for IPEC-J2. Bacterial and yeast cells were harvested by centrifugation, resuspended in DMEM, and adjusted to approximately 1.0 × 10^9^ CFU/mL. An aliquot (1.0 × 10^8^ CFU/well) was added to each well and incubated at 37 °C under 5% CO₂ for 2 h. After incubation, non-adherent cells were removed by washing three times with sterile PBS. Adherent cells were then released using 1% Triton X-100 (Sigma-Aldrich, USA) and quantified using LAB Petrifilm and RYM Petrifilm for bacterial and yeast strains, respectively (3 M, USA). *L. rhamnosus* GG (ATCC 53103) was used as a reference strain.

### 2.3 Antibiotic susceptibility of bacterial strains

The antibiotic susceptibility of *Pediococcus* and *Lactobacillus* strains was evaluated using E-test strips (bioMérieux, Marcy-l’Étoile, France) against ampicillin, vancomycin, gentamicin, kanamycin, streptomycin, erythromycin, clindamycin, tetracycline, and chloramphenicol. Fresh bacterial cultures were spread onto de Man, Rogosa and Sharpe (MRS) agar plates, and E-test strips were applied to the agar surface before incubation at 37 °C for 24 h. The minimum inhibitory concentration (MIC) values were determined according to the manufacturer’s instructions and interpreted based on the microbiological cutoff values established by the European Food Safety Authority (EFSA) guidelines (15).

### 2.4 Antifungal susceptibility of yeast strain

The antifungal susceptibility of *Saccharomyces cerevisiae* CACC699 was evaluated using E-test strips (bioMérieux, France) against voriconazole, amphotericin B, fluconazole, and itraconazole. Fresh yeast cultures were spread onto yeast malt (YM) agar plates, and E-test strips were applied to the agar surface before incubation at 30 °C for 24 h. The minimum inhibitory concentration (MIC) values were determined according to the manufacturer’s instructions and interpreted based on the epidemiological cutoff values (ECOFFs) established by the European Committee on Antimicrobial Susceptibility Testing (EUCAST) guidelines (16, 17).

### 2.5 Biogenic amine production assay

Biogenic amine (BA) production was determined following the method described by Zhang et al. (18). Briefly, 10 mL of bacterial culture was mixed with 40 mL of 0.1 N HCl and extracted for 30 min. The mixture was centrifuged at 7,000 × g for 15 min at 4 °C, and the supernatant was filtered. The filtered extract was mixed with 0.5 mL of saturated sodium carbonate solution and 0.8 mL of 1% dansyl chloride solution as a derivatization reagent. The mixture was incubated at 45 °C for 1 h for derivatization. Subsequently, 0.5 mL of 10% proline solution and 5 mL of ether were added, followed by mixing. The organic phase was collected and evaporated, and the residue was dissolved in acetonitrile. The solution was centrifuged at 4,050 × g for 5 min, and the supernatant was filtered through a 0.22 μm PVDF syringe filter prior to analysis. Biogenic amines were quantified using high-performance liquid chromatography (HPLC; Waters, USA). Standard amines (putrescine, cadaverine, histamine, spermidine, and spermine) were obtained from Sigma-Aldrich.

### 2.6 Bacterial and yeast strains and solid-state fermentation

In this study, *P. pentosaceus* CACC616, *L. reuteri* CACC607, *L. dextrinicus* CACC889, *L. pentosus* CACC891, and *S. cerevisiae* CACC699 were used for the preparation of the microbial bioactive complex (MBC). These strains were originally isolated from the feces of healthy pigs in South Korea and initially selected based on their probiotic potential. Prior to MBC preparation, a preliminary solid-state fermentation screening was conducted using seven candidate lactic acid bacterial strains in four candidate formulations. Candidate formulations were evaluated based on viable cell counts after fermentation, pH changes during fermentation, and resistance to fungal contamination during storage. Based on overall fermentation performance and stability, a consortium consisting of *P. pentosaceus* CACC616, *L. reuteri* CACC607, *L. dextrinicus* CACC889, and *L. pentosus* CACC891 was selected. *S. cerevisiae* CACC699 was subsequently incorporated to improve fermentation stability, minimize the risk of undesirable yeast contamination during solid-state fermentation, and promote the production of fermentation-derived metabolites, resulting in the final MBC formulation used in this study. Solid-state fermentation (SSF) was performed to enhance microbial viability and generate fermentation-derived metabolites under low-moisture conditions. The fermentation substrate consisted of 60% wheat bran, 20% soybean meal, and 20% distillers dried grains with solubles (DDGS), supplemented with 1% glucose, 1% lactose, 1% yeast extract, and 0.5% molasses. Water was added to achieve a final moisture content of 60%. For inoculation, microbial cultures were added at a total volume of 3.0% (v/w) of the dry substrate (3.0 mL per 100 g substrate). The inoculum consisted of *P. pentosaceus* CACC616, *L. reuteri* CACC607, *L. dextrinicus* CACC889, *L. pentosus* CACC891, and *S. cerevisiae* CACC699 at a ratio of 0.5:0.5:0.5:0.5:1.0, respectively, corresponding to a bacterial-to-yeast ratio of approximately 2:1. Prior to inoculation, lactic acid bacterial cultures and the yeast culture were prepared at viable cell densities of approximately 5 × 10^6^ and 5 × 10^5^ CFU/mL, respectively. The inoculum was thoroughly mixed with the substrate until homogeneous distribution was achieved. No additional water was added during inoculation beyond the moisture naturally present in the substrate ingredients. Fermentation was conducted at 30 °C for 24 h. After fermentation, the substrate was dried at 50 °C for 3 h, and viable cell counts, nutrient composition, and SCFA contents were subsequently determined as described in Section 2.7. The final MBC product contained approximately 2.4 × 10^9^ CFU/g of lactic acid bacteria and 5.17 × 10^6^ CFU/g of yeast. The MBC product was incorporated into the basal diet at an inclusion rate of 0.5%.

### 2.7 Nutrient composition and SCFAs of the MBC product

After solid-state fermentation (SSF), the nutrient composition of the MBC product, including moisture, crude protein, crude fat, ash, fiber, neutral detergent fiber (NDF), and acid detergent fiber (ADF), was determined by the Korea Feed Ingredients Association (KFIA) according to the Korean Standard Methods for Feed Analysis (19). The concentrations of acetic acid, lactic acid, and propionic acid in the MBC product were also analyzed by the Korea Feed Ingredients Association (KFIA) according to the same standard methods using high-performance liquid chromatography (HPLC) (19).

### 2.8 Fecal short chain fatty acid analysis

Fecal samples were collected to determine short-chain fatty acid (SCFA) concentrations. Fecal SCFA analysis was performed using samples from 15 piglets per group, based on sample availability. Approximately 50 mg of fecal sample was extracted with 10 mL of 50% (v/v) acetonitrile by shaking at 2,500 rpm for 10 min. The mixture was centrifuged at 4,000 rpm for 10 min, and the supernatant was collected. For derivatization, 100 μL of the supernatant was mixed with 40 μL of 200 mM 3-nitrophenylhydrazine (3-NPH), followed by the addition of 40 μL of 120 mM 1-ethyl-3-(3-dimethylaminopropyl) carbodiimide (EDC). The mixture was incubated at 40 °C for 30 min, and the reaction was terminated by adding 400 μL of 0.1% formic acid. The final solution was diluted with 10% acetonitrile prior to analysis. SCFAs, including acetic acid, propionic acid, butyric acid, lactic acid, valeric acid, and isovaleric acid, were quantified using a UPLC–MS/MS system (ACQUITY UPLC I-Class/Xevo TQ-S micro, Waters, USA) equipped with an electrospray ionization (ESI) source operating in negative ion mode. Chromatographic separation was achieved using an ACQUITY UPLC BEH C18 column (2.1 × 100 mm, 1.7 μm; Waters, USA). The mobile phase consisted of water (A) and acetonitrile (B), both containing 0.1% formic acid, delivered at a flow rate of 0.5 mL/min under gradient conditions. Quantification was performed in multiple reaction monitoring (MRM) mode using the following transitions (Q1/Q3, m/z): acetic acid (194.00/137.00), propionic acid (208.00/137.00), butyric acid (220.00/137.00), lactic acid (224.13/137.00), valeric acid (236.00/137.00), and isovaleric acid (236.00/137.00).

### 2.9 Animal diet and experimental design

The experiment was conducted at a commercial swine farm located in Boseong County, South Jeolla Province, South Korea. A total of 34 weaned piglets (Duroc × Landrace × Yorkshire) were used in this study. Piglets were weaned at 21 days of age (initial body weight: 7.2 ± 0.16 kg), and the experimental diets were administered immediately after weaning. Animals were balanced by sex. Animals were randomly assigned to two groups, with 17 piglets allocated to the MBC group and the remaining to the control (CON) group. All piglets were fed a basal diet consisting of a commercial feed (Cargill Agri Purina, Inc., South Korea). The feeding program consisted of Purina Neo Smile 2 during days 1–9, Purina Neo Smile 3 during days 10–20, and Purina Relay during days 21–33. The nutrient composition of the diets was described by Park et al. (20). During the experimental period, piglets had ad libitum access to feed and water, and feed intake was recorded daily on a pen basis based on the amount of feed offered and refused. Individual body weights (BW) were measured on day 0 (experimental start) and day 33 (experimental end). Average daily gain (ADG) was calculated for each piglet, whereas average daily feed intake (ADFI) was determined on a pen basis. Feed conversion ratio (FCR) was calculated by dividing pen-based ADFI by individual ADG and was expressed at the individual level. Because this study was conducted under commercial farm conditions, feed intake could only be measured at the pen level, whereas body weight was recorded individually. Therefore, ADFI was calculated on a pen basis, and the derived FCR values should be interpreted as estimates of feed conversion performance under practical production conditions. All piglets were monitored for signs of diarrhea and mortality throughout the 33-day experimental period.

### 2.10 Sample collection, sequencing, and data processing

Rectal swab samples (BD BBL CultureSwab, BD, USA) were collected from 34 weaned piglets on days 0 and 33. Microbial genomic DNA (gDNA) was extracted from the rectal swab samples according to a previously described method (21). The extracted gDNA was quantified using a DropSense96 spectrophotometer (Trinean NV, Belgium). Amplicon libraries targeting the bacterial 16S rRNA gene were prepared using the MGIEasy Universal Library Prep Kit (MGI, China) and 300-bp paired-end sequencing was performed on the MGISEQ-2000 platform (LAS, South Korea).

### 2.11 Microbiome diversity, composition, and correlation analysis

The QIIME2 (22) DADA2 package [version 2019.4.0; Callahan et al. (23)] was used to denoise the paired end sequences, duplicate them, merge forward and reverse reads, and filter chimeras. Representative sequences were classified using QIIME2 scikit-learn [version 2019.4.0; Pedregosa et al. (24)] with a pretrained classifier trained on the SILVA [database release 132; Quast et al. (25)], and multiple sequence alignments were conducted using QIIME2 MAFFT [version 2019.4.0; Katoh et al. (26)]. Downstream data analysis was conducted in the R statistical environment (27) using a combination of custom scripting with the microbiome, phyloseq, vegan, and ggplot2 packages. Relative abundance was analyzed using the phyloseq [version 1.46.0; McMurdie et al. (28)] and microbiome [version 1.24.0; Lahti and Shetty (29)] packages in R. *α*-diversity indices, including the Chao1 richness estimator (30), Shannon index (31), and Simpson index (32), were calculated using the phyloseq package in R. *β*-diversity (33), which measures the dissimilarity of the microbial community composition between samples, was characterized using the Bray–Curtis index. A principal coordinate analysis (PCoA) plot was used to visualize the Bray–Curtis dissimilarity among the samples. Spearman’s correlation analysis was performed at the genus level to evaluate associations between microbial taxa and experimental variables.

### 2.12 Statistical analysis

All statistical analyses were performed using GraphPad Prism version 8.0 (GraphPad Software, Inc., USA) and R software. Data are presented as the mean ± standard error of the mean (SEM) or ± standard deviation (SD), and statistical significance was set at *p* < 0.05. Normality was assessed using the Shapiro–Wilk test. For growth performance and SCFA data that satisfied the assumption of normality, comparisons between two groups were performed using an unpaired *t*-test. For comparisons involving more than two groups, one-way analysis of variance (ANOVA) followed by Tukey’s *post hoc* test was applied. For microbial taxa that did not meet the assumption of normality, non-parametric methods were used. Differences in relative microbial abundances between groups were assessed using the Mann–Whitney test. Alpha diversity indices were analyzed using the Kruskal–Wallis test. Beta diversity was evaluated based on Bray–Curtis dissimilarity using principal coordinate analysis (PCoA) with the vegan package (v.2.6–8) in R. Statistical significance of beta diversity was assessed using permutational multivariate analysis of variance (PERMANOVA).

## 3 Results

### 3.1 Probiotic characterization and safety assessment of the selected strains

**Figure 1:**
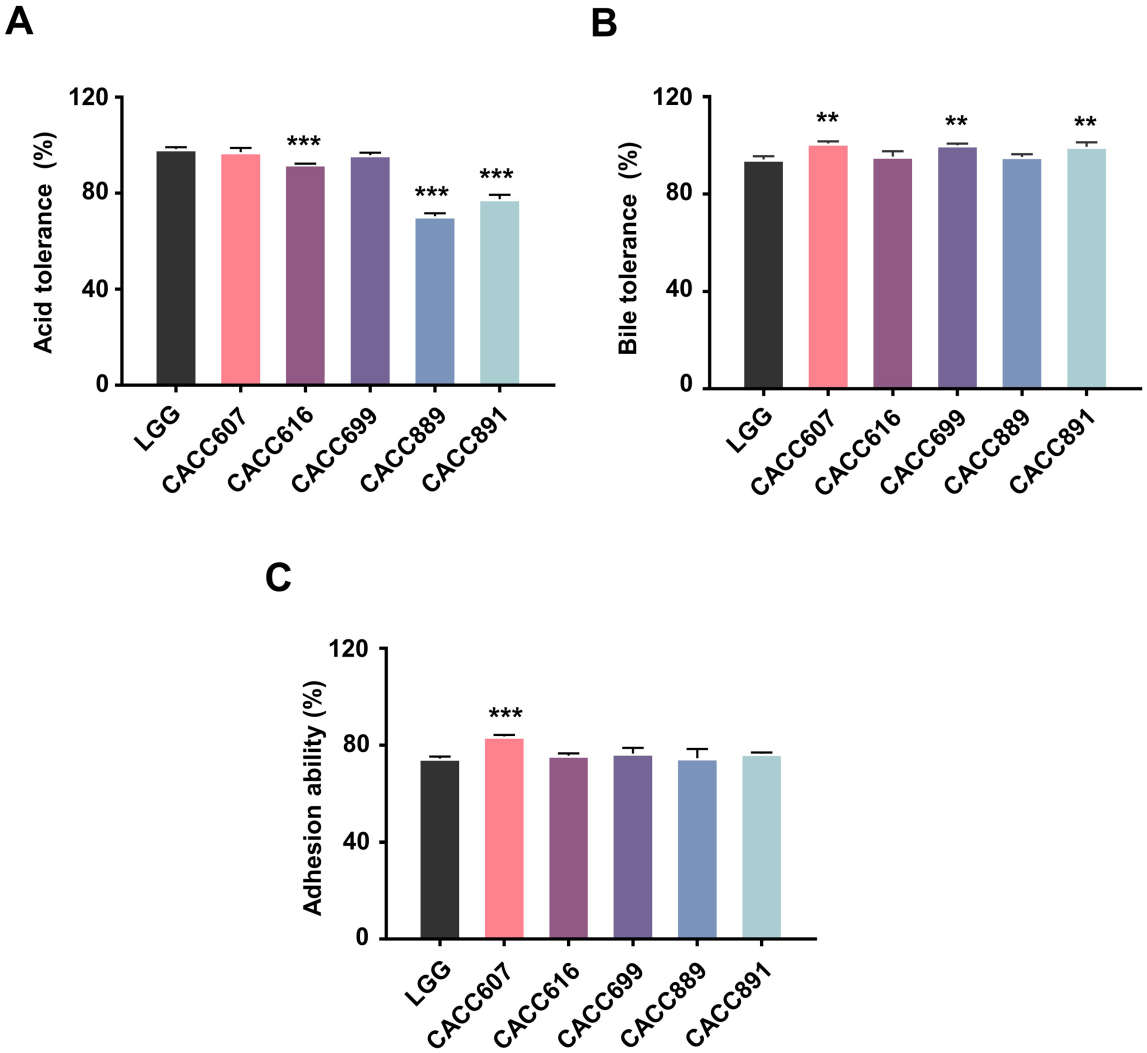
Probiotic characteristics of the selected strains. **(A)** Acid tolerance, **(B)** Bile tolerance and **(C)** Adhesion to porcine intestinal epithelial cells (IPEC-J2) of *Lactobacillus reuteri* CACC607, *Pediococcus pentosaceus* CACC616, *Saccharomyces cerevisiae* CACC699, *L. dextrinicus* CACC889, and *L. pentosus* CACC891. *L. rhamnosus* GG was used as a reference probiotic strain. Data are presented as means ± SD. ***p < 0.001; **p < 0.01.

All selected strains maintained high viability under simulated gastrointestinal conditions and exhibited acid and bile salt tolerance comparable to or greater than that of the reference probiotic strain, *L. rhamnosus* GG (LGG) (Figure 1). LGG was used as a reference strain because it is one of the most extensively studied and well-established probiotic strains and is widely recognized as a benchmark strain in probiotic characterization studies due to its documented acid and bile tolerance, adhesion capacity, and safety profile. The selected isolates also demonstrated adhesion abilities comparable to LGG, indicating their potential to persist within the gastrointestinal tract and interact with the intestinal epithelium (Figure 1C). The safety characteristics of the selected strains were further evaluated through antibiotic and antifungal susceptibility testing, hemolytic activity assessment, and biogenic amine production analysis. *P. pentosaceus* CACC616, *L. reuteri* CACC607, *L. dextrinicus* CACC889, and *L. pentosus* CACC891 were all susceptible to ampicillin (Table 1). All candidate strains exhibited high vancomycin MIC values, ranging from ≥129 to ≥256 μg/mL. However, susceptibility to vancomycin is not required by the European Food Safety Authority (EFSA; 15) for the safety assessment of lactic acid bacteria. Although several MIC values exceeded the established microbiological breakpoints for certain antibiotics, the observed resistance patterns were consistent with intrinsic resistance characteristics commonly reported in lactic acid bacteria (Table 1). The antifungal susceptibility of *S. cerevisiae* CACC699 was determined according to the CLSI M27-A guideline (16). Because clinical breakpoints have not been established for *S. cerevisiae*, epidemiological cutoff values (ECOFFs) were applied according to EUCAST recommendations (17). *S. cerevisiae* CACC699 was susceptible to fluconazole and itraconazole but resistant to voriconazole and amphotericin B, with ECOFF values of ≤0.125 μg/mL for voriconazole and ≤0.5 μg/mL for amphotericin B (Table 2). Hemolytic activity testing revealed that all selected strains exhibited *γ*-hemolysis, characterized by the absence of red blood cell lysis and no visible hemolytic zone surrounding colonies on blood agar plates (Supplementary Figure S1). No *α*- or *β*-hemolytic activity was observed. These results indicate that the selected strains do not possess hemolytic activity and support their safety for potential probiotic application. Biogenic amine production was generally low or undetectable among the tested strains (Table 3). Histamine production was not detected in any strain. *L. reuteri* CACC607 and *L. dextrinicus* CACC889 did not produce detectable levels of any tested biogenic amines. Low concentrations of putrescine, cadaverine, spermidine, and spermine were detected in *P. pentosaceus* CACC616, *S. cerevisiae* CACC699, and *L. pentosus* CACC891, with total biogenic amine concentrations ranging from 11.05 to 31.49 ppm (Table 3). These levels were considered low and unlikely to raise safety concerns under the intended conditions of use. These results demonstrate that the selected bacterial and yeast strains possess desirable probiotic characteristics, including tolerance to gastrointestinal conditions, adhesion ability, and favorable safety profiles, supporting their suitability for inclusion in the microbial bioactive complex (MBC).

**Table 1:** Antibacterial susceptibility profiles of bacterial strains.

Antibiotics	MIC (μg/mL)			
	*L. reuteri* CACC607	*P. pentosaceus* CACC616	*L. dextrinicus* CACC889	*L. pentosus* CACC891
Ampicillin	0.02	3	1.5	0.125
Vancomycin	≥129	≥ 256	≥ 256	≥256
Gentamicin	4	**32**	**≥ 256**	**24**
Kanamycin	**≥ 256**	**≥ 256**	**≥ 256**	**≥256**
Streptomycin	**96**	**384**	**≥ 1,024**	48
Erythromycin	**≥ 256**	1	**3**	1
Clindamycin	**≥ 256**	0.094	**24**	0.75
Tetracycline	**≥ 256**	**24**	**64**	32
Chloramphenicol	**12**	**6**	**32**	6

MIC, minimum inhibitory concentrations; in bold, MIC values higher than the microbiological breakpoints defined by the EFSA panel [EFSA panel on additives and products or substances used in animal feed (FEEDAP), 2018].

**Table 2:** Antifungal susceptibility of the CACC699 yeast strain.

Antifungal agent	MIC (μg/mL)
Voriconazole	**0.13**
Amphotericin	**1.3**
Fluconazole	8
Itraconazole	1.0

MIC, minimum inhibitory concentrations; in bold, MIC values higher than the microbiological breakpoints defined by the EUCAST guidelines.

**Table 3:** Determination of biogenic amine production by the strains.

Parameter	BA-producing ability (ppm)				
	CACC607	CACC616	CACC699	CACC889	CACC891
Putrescine	ND	5.19	4.28	ND	ND
Cadaverine	ND	2.59	2.36	ND	2.32
Histamine	ND	ND	ND	ND	ND
Spermidine	ND	11.5	18.9	ND	5.19
Spermine	ND	5.45	5.95	ND	3.54
Total	ND	24.73	31.49	ND	11.05

BA, biogenic amine; ND, not detected; CACC607, *L. reuteri* CACC607; CACC616, *P. pentosaceus* CACC616; CACC699, *S. cerevisiae* CACC699; CACC889, *L. dextrinicus* CACC889; CACC891, *L. pentosus* CACC891.

### 3.2 Characteristics of the solid-state fermentation product

Solid-state fermentation (SSF) of the five selected strains was successfully performed, resulting in substantial increases in viable microbial populations, with lactic acid bacteria and yeast increasing by 186-fold and 21-fold, respectively. The SSF process resulted in a decrease in pH and promoted the accumulation of fermentation-derived metabolites, including lactic acid and acetic acid (Table 4). These changes indicate active microbial fermentation and successful establishment of the selected microbial consortium within the substrate. The increased viable cell counts and production of organic acids demonstrate that the SSF process effectively generated a stable microbial bioactive complex suitable for dietary supplementation.

**Table 4:** Nutritional composition of the microbial bioactive complex (MBC) before and after solid-state fermentation.

Parameter	Fermentation	
	0 h	24 h
pH	5.8	4.74
Moisture (%)	43.5	44.4
Crude protein (%)	14.5	14.91
Crude fat (%)	2.2	2.3
Crude ash (%)	3.34	2.47
Crude fiber (%)	4.83	4.39
NDF (%)	17.71	17.12
ADF (%)	5.25	6.02
Propionic acid (g/kg)	ND	ND
Lactic acid (g/kg)	0.962	1.295
Acetic acid (g/kg)	0.325	1.896
Lactic acid bacteria (CFU/g)	1.29 × 10^7^	2.4 × 10^9^
Yeast (CFU/g)	2.45 × 10^5^	5.17 × 10^6^

NDF, neutral detergent fiber; ADF, acid detergent fiber; ND, not detected. Values were obtained from the official analytical report issued by an accredited feed analysis laboratory, the Korea Feed Ingredients Association (KFIA), and therefore are presented as certified values rather than mean ± SEM. Replicate measurements and corresponding SEM values were not available from the analytical report.

### 3.3 Effect of supplemental feed on growth performance and blood parameters

The initial body weight (BW) of piglets did not differ between the control (7.3 ± 0.1 kg) and MBC groups (7.0 ± 0.2 kg) (*p* > 0.05; Table 5). Similarly, final BW was not significantly different between groups (control: 20.6 ± 0.6 kg; MBC: 19.0 ± 0.5 kg; Table 5). Average daily gain (ADG) showed a similar pattern, with slightly higher values in the control group (401.8 ± 18.6 g) compared with the MBC group (363.8 ± 16.3 g), although the difference was not statistically significant (*p* > 0.05; Table 5). In contrast, average daily feed intake (ADFI) was significantly lower in the MBC group (537 ± 4.9 g) than in the control group (661 ± 5.4 g) (*p* < 0.05; Table 5). Despite the reduced feed intake, feed conversion ratio (FCR) was significantly improved in the MBC group compared with the control (*p* < 0.05), indicating enhanced feed utilization efficiency (Table 5). Blood parameters did not differ significantly between the MBC and control groups (*p* > 0.05; Supplementary Figure S2). No mortality was observed during the study, and no severe diarrhea requiring veterinary intervention was recorded in either the CON or MBC group.

**Table 5:** Effects of dietary microbial bioactive complex (MBC) supplementation on growth performance in weaned piglets.

**Parameter (0-33d)**	**CON**	**MBC**
Initial BW (kg)	7.3 ± 0.1^a^	7.0 ± 0.2^a^
Final BW (kg)	20.6 ± 0.6^a^	19.0 ± 0.5^a^
ADG (g)	401.8 ± 18.6^a^	363.8 ± 16.3^a^
ADFI (g)	661 ± 5.4^a^	537 ± 4.9^b^
FCR	1.7 ± 0.0^a^	1.5 ± 0.0^b^

^a,b^Values within the same row with different superscripts differ significantly (*P* < 0.05). Data are expressed as mean ± SEM (*n* = 17). ADG, average daily gain; ADFI, average daily feed intake; FCR, feed conversion ratio; MBC, diet supplemented with a microbial bioactive complex produced via solid-state fermentation and containing strains of *Pediococcus*, *Lactobacillus*, and *Saccharomyces*.

### 3.4 Comparison of gut microbiota community diversity and composition

**Figure 2:**
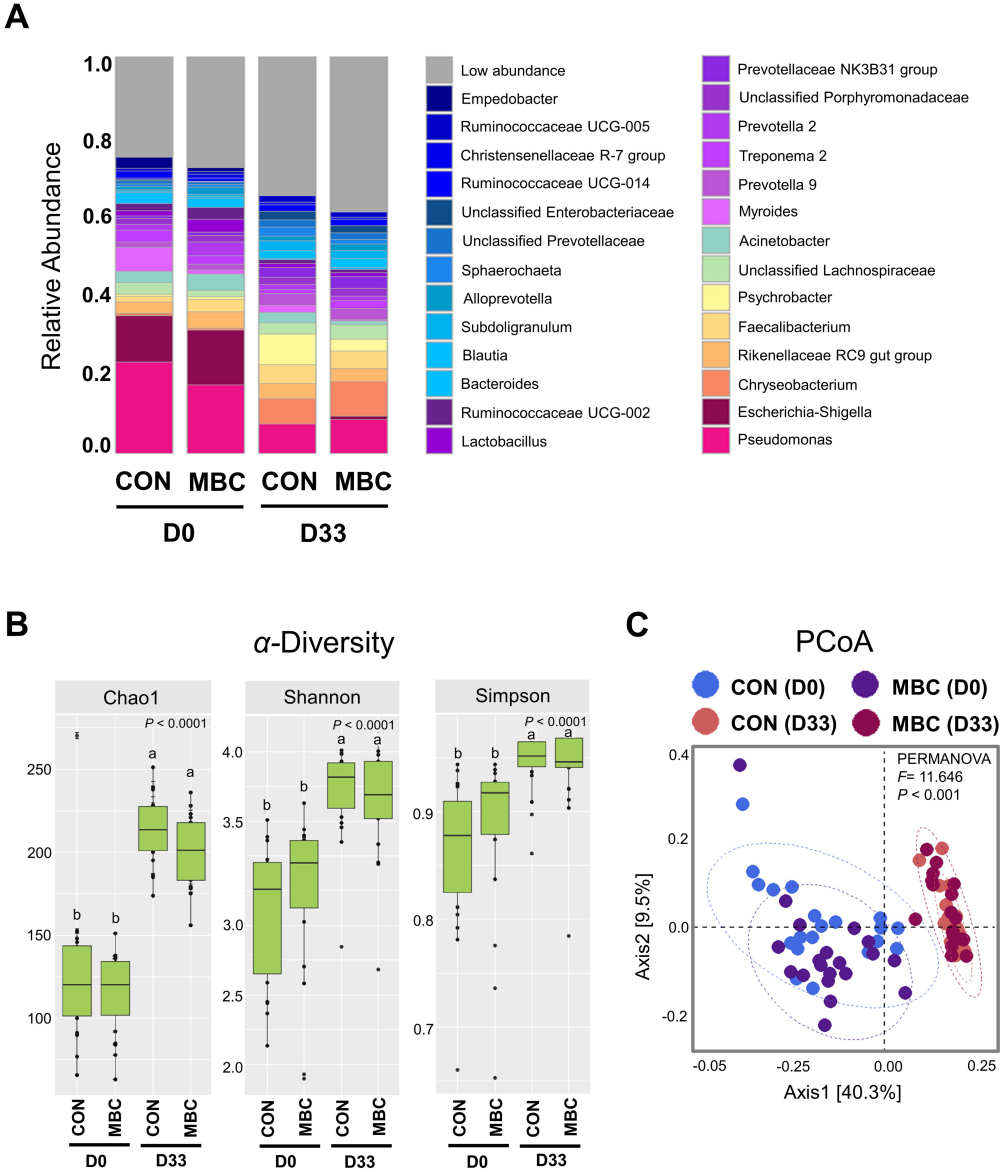
Effects of microbial bioactive complex (MBC) supplementation on gut microbiota composition and diversity in weaned piglets. **(A)** Relative abundance of bacterial taxa at the genus level in the control (CON) and MBC groups at day 0 (D0) and day 33 (D33). Only major taxa are shown, and low-abundance taxa are grouped as “low abundance.” **(B)** Alpha diversity indices (Chao1 richness, Shannon diversity, and Simpson index) of fecal microbiota. **(C)** Principal coordinate analysis (PCoA) based on Bray–Curtis dissimilarity showing differences in microbial community structure among groups and time points. Statistical significance for alpha diversity was determined using the Kruskal–Wallis test, followed by pairwise comparisons using the Wilcoxon rank-sum test. Values with different superscripts (A,B) indicate significant differences (*p* < 0.05).

**Figure 3:**
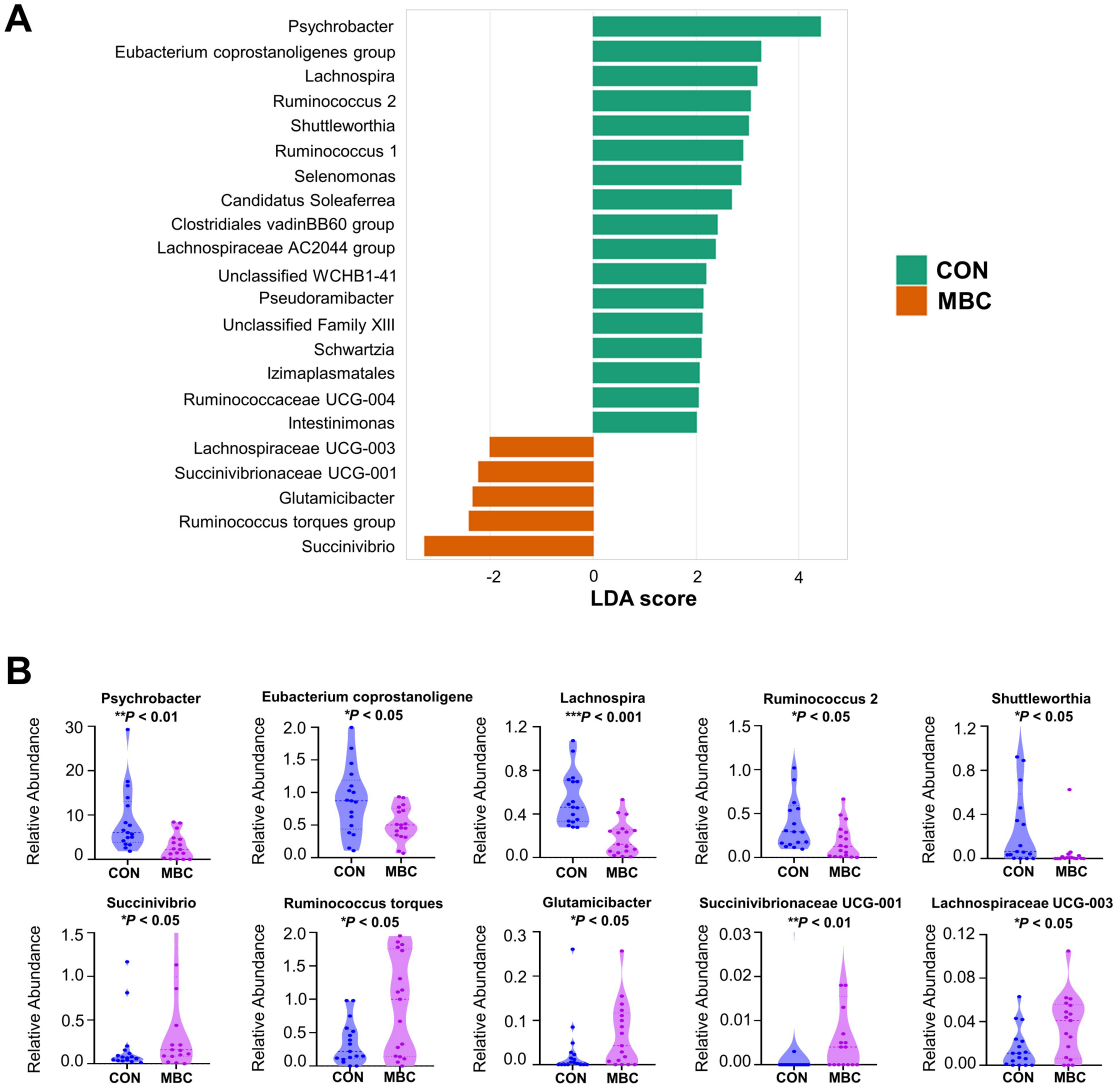
Differential microbial taxa in response to microbial bioactive complex (MBC) supplementation **(A)** Linear discriminant analysis effect size (LEfSe) identifying differentially abundant taxa between the control (CON) and MBC groups. Only taxa with an LDA score > 2.0 are shown. **(B)** Relative abundances of the top five bacterial genera enriched in each group (CON and MBC), as identified by LEfSe analysis.

To investigate the effects of MBC supplementation on intestinal microbiota diversity and composition in weaned piglets, 16S rRNA gene amplicon sequencing was performed using fecal samples collected on days 0 and 33 (Figure 2). Alpha diversity indices (Chao1, Shannon, and Simpson; Figure 2B) showed significant differences between days 0 and 33, indicating temporal changes in microbial richness and diversity. However, no significant differences were observed between the control and MBC groups. To further evaluate microbial community structure, principal coordinate analysis (PCoA) based on Bray–Curtis dissimilarity was performed (Figure 2C). The results revealed clear temporal shifts in microbial community composition between days 0 and 33, although no distinct separation was observed between the control and MBC groups. Linear discriminant analysis effect size (LEfSe) was performed to identify microbial taxa associated with MBC supplementation (Figure 3A). Several taxa, including *Psychrobacter*, *Eubacterium coprostanoligenes* group, *Lachnospira*, *Ruminococcus 2*, and *Shuttleworthia*, were significantly decreased in the MBC group compared with the control (*p* < 0.05). In contrast, the relative abundances of *Succinivibrio*, *Ruminococcus torques* group, *Glutamicibacter*, *Succinivibrionaceae UCG-001*, and *Lachnospiraceae UCG-003* were significantly increased in the MBC group (*p* < 0.05; Figure 3B; Supplementary Table S1).

### 3.5 Effect of potential MBC supplemental feed on fecal SCFAs

Dietary supplementation with MBC significantly altered fecal short-chain fatty acid (SCFA) profiles (Table 6). The proportion of propionic acid was significantly increased in the MBC group (*p* < 0.05; Table 6), whereas the relative proportion of butyric acid was significantly decreased (*p* < 0.05; Table 6). The proportion of acetic acid showed an increasing trend in the MBC group, although this difference was not statistically significant (*p* > 0.05; Table 6). These results indicate that MBC supplementation modulates gut microbial fermentation patterns, characterized by a shift in SCFA composition toward a higher propionate proportion despite an overall reduction in total SCFA concentrations.

**Table 6:** Effects of dietary microbial bioactive complex (MBC) supplementation on fecal SCFAs in weaned piglets.

Parameter		CON	MBC
SCFAs (% of total) (ppm in parentheses)	Acetic acid	38.8^a^ (6,723 ± 50)	42.5^a^ (6,234 ± 104)
	Propionic acid	24.4^a^ (4,271 ± 54)	28.3^b^ (4,408 ± 127)
	Butyric acid	26.4^a^ (4,650 ± 73)	20.6^b^ (3,124 ± 72)
	Lactic acid	1.6^a^ (266 ± 16)	0.8^a^ (117 ± 5)
	Valeric acid	6.7^a^ (1,202 ± 31)	6.0^a^ (991 ± 47)
	isovaleric acid	2.1^a^ (375 ± 8)	1.8^a^ (283 ± 8)

^a,b^Values within the same row with different superscripts differ significantly (*p* < 0.05). Relative proportions (%) were calculated based on total SCFAs. Superscripts indicate significant differences based on relative proportions (%). Data are expressed as mean ± SEM (*n* = 15). SCFAs, short chain fatty acids; CON, control diet; MBC, diet supplemented with a microbial bioactive complex produced via solid-state fermentation and containing strains of *Pediococcus*, *Lactobacillus*, and *Saccharomyces*.

### 3.6 Correlation between gut microbiota, SCFAs, MBC treatment, and growth performance

**Figure 4:**
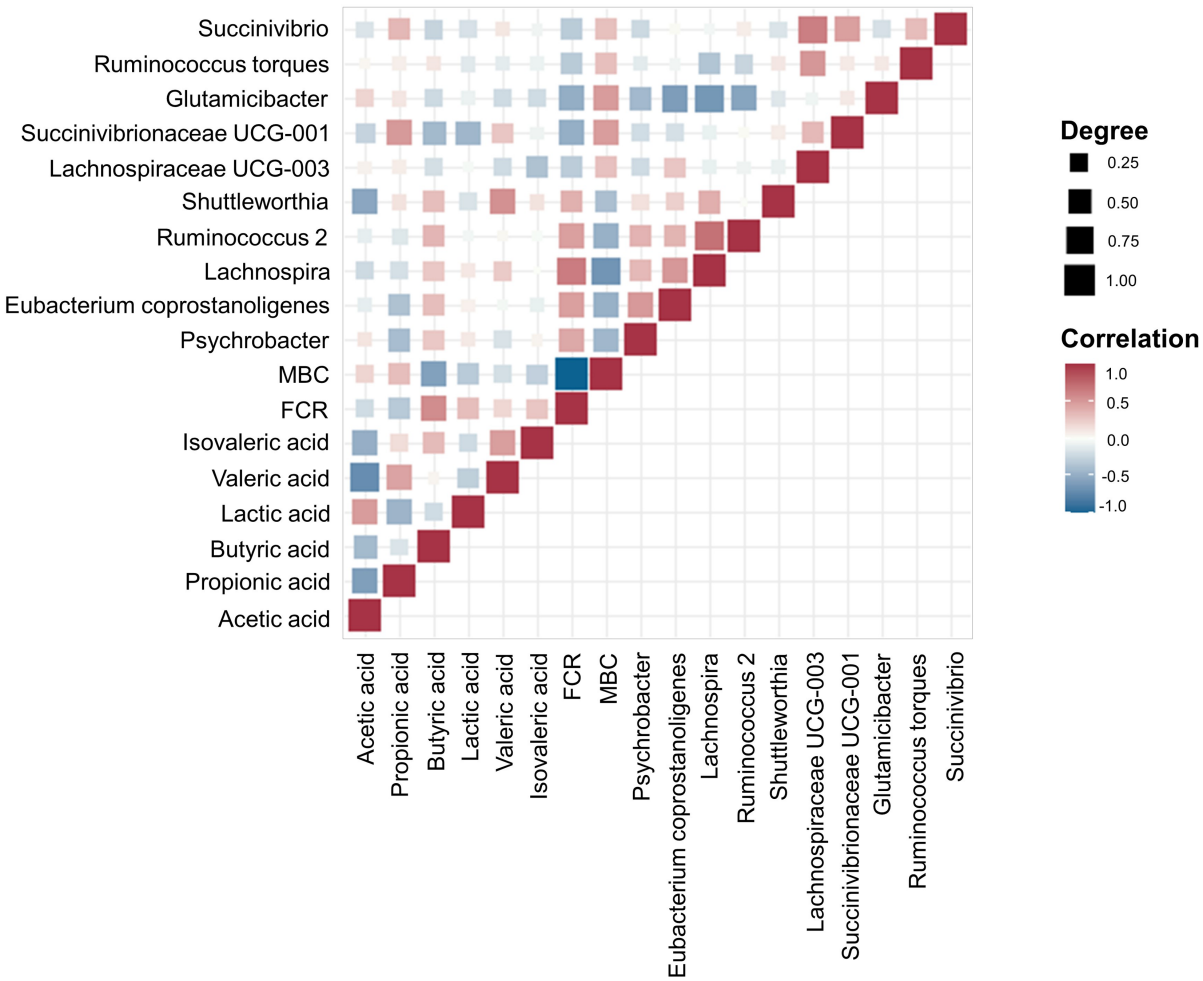
Associations among gut microbiota, short chain fatty acids (SCFAs), and growth performance correlation matrix showing associations among microbial taxa, SCFA profiles, MBC treatment, and feed conversion ratio (FCR). Correlations were calculated using Spearman’s rank correlation coefficients. Correlation coefficients range from −1 to 1, with the strongest positive (*r* = 1) and negative (*r* = −1) correlations indicated in red and blue, respectively. Color intensity and square size correspond to the magnitude of the correlation.

To further investigate the relationships among microbial taxa, SCFA profiles, MBC treatment, and growth performance, correlation analysis was performed (Figure 4), with MBC treatment included as a categorical variable. Notably, MBC treatment was positively associated with taxa enriched in the MBC group, including *Succinivibrio* and *Succinivibrionaceae* UCG-001, and was positively correlated with the proportion of propionic acid while being negatively correlated with feed conversion ratio (FCR). In contrast, taxa reduced in the MBC group, such as *Psychrobacter*, *Eubacterium coprostanoligenes* group, and *Lachnospira*, were positively associated with butyric acid and FCR but negatively associated with propionic acid and MBC treatment. Collectively, these findings indicate that MBC treatment is associated with coordinated shifts in microbial taxa and SCFA profiles, particularly the enrichment of propionate-associated taxa.

## 4 Discussion

The intestinal health and feed efficiency of weaned piglets are key determinants of economic performance in swine production (1, 34, 35). Accordingly, microbial-based feed additives have been widely investigated as nutritional strategies to stabilize gut function and improve production efficiency (6, 36, 37). In addition to conventional probiotic preparations composed solely of viable microorganisms, increasing attention has been directed toward microbial bioactive complexes that integrate live cells with fermentation-derived metabolites and secondary compounds (37–43). Such systems may exert synergistic effects on nutrient utilization and intestinal fermentation dynamics. In the present study, the successful production of a multi-strain microbial bioactive complex via solid-state fermentation, characterized by increased viable microbial populations and fermentation-derived metabolites, supports its potential as a functional feed additive. Dietary supplementation with the microbial bioactive complex significantly improved feed conversion ratio (FCR) while reducing average daily feed intake (ADFI), without affecting final body weight or average daily gain (ADG). These findings suggest enhanced feed utilization efficiency rather than increased growth rate. Previous studies have reported improvements in both ADG and FCR following supplementation with fermented feed products (44, 45); however, responses vary depending on microbial composition, fermentation conditions, inclusion level, and diet formulation (46, 47). The reduction in FCR observed in this study is consistent with reports indicating that fermentation-derived metabolites may enhance nutrient availability and digestive efficiency (48). Therefore, the improved feed efficiency may reflect enhanced metabolic utilization of nutrients rather than increased feed intake or growth potential. With respect to gut microbial ecology, fermented feed and probiotic supplementation have been reported to influence microbial diversity and suppress pathogenic bacteria while supporting intestinal homeostasis (49–51). In the present study, no significant differences in alpha or beta diversity indices were observed between treatments, consistent with previous reports (52). Although pronounced diversity shifts have been described in other studies (53), microbial responses appear to depend on host age, diet composition, and environmental conditions. The absence of broad diversity changes suggests that MBC supplementation induced selective modulation of specific microbial taxa rather than large-scale restructuring of the gut microbiota. Notably, MBC supplementation was associated with enrichment of specific taxa, including *Succinivibrio* and *Succinivibrionaceae UCG-001*, which are known to be involved in carbohydrate fermentation and succinate/propionate production. In contrast, taxa such as *Psychrobacter*, *Eubacterium coprostanoligenes* group, and *Lachnospira* were reduced following supplementation. These changes suggest a shift in microbial metabolic potential rather than overall diversity, indicating targeted ecological modulation. Consistent with these microbial changes, fecal SCFA profiles were significantly altered by MBC supplementation. The relative proportion of propionic acid was significantly increased. Propionate is a key end-product of carbohydrate fermentation and has been associated with improved energy utilization and metabolic efficiency in the host. Correlation analysis further supported this interpretation, demonstrating that MBC treatment was positively associated with propionate-related taxa and negatively associated with FCR. These results indicate coordinated interactions between microbial composition and fermentation metabolites, suggesting that MBC supplementation promotes a microbial–metabolic profile linked to improved feed efficiency. Taken together, the present study suggests that microbial bioactive complex supplementation is associated with altered gut microbial fermentation patterns and improved feed conversion-related parameters in weaned piglets. Rather than inducing broad changes in microbial diversity, MBC appeared to selectively modulate specific microbial taxa and metabolic outputs, particularly those associated with propionate production. These findings highlight the potential importance of functional microbial shifts in shaping host metabolic responses. Several limitations of the present study should be acknowledged. Feed intake was measured on a pen basis under commercial farm conditions, whereas body weight gain was measured individually. Consequently, the calculated FCR values should be interpreted cautiously, as individual feed intake could not be directly quantified. Therefore, the observed reduction in FCR should be regarded as an indicator of feed conversion performance under practical production conditions rather than definitive evidence of improved nutrient utilization. In addition, nutrient digestibility and energy utilization were not evaluated, limiting interpretation of the biological mechanisms underlying the observed feed conversion responses. The MBC product was generated through solid-state fermentation and therefore contained not only viable microorganisms but also fermentation-derived metabolites and residual fermented substrate components. Therefore, the observed changes in fecal SCFA profiles may reflect the combined effects of the supplemented microorganisms, fermentation-derived products, and microbiota-associated fermentation processes. Although propionic acid was not detected in the MBC product itself, the increased relative proportion of fecal propionate may have been associated with alterations in the intestinal microbial ecosystem, including enrichment of taxa related to succinate and propionate production. Nevertheless, the relative contributions of the supplemented microorganisms, fermentation-derived metabolites, and residual fermented substrate components to the observed microbial and metabolic responses cannot be fully distinguished in the present study. Strain-specific analyses were not performed to directly verify the survival, persistence, or colonization of the administered microorganisms in the gastrointestinal tract. Therefore, the observed changes in gut microbial composition should be interpreted as associations with MBC supplementation rather than direct evidence of colonization by the supplemented strains. Future studies incorporating evaluations of intestinal morphology, epithelial barrier-associated proteins, and inflammatory cytokines would provide deeper insight into how the observed microbial and metabolic changes are directly related to intestinal health and host physiological responses. Accordingly, although significant associations were identified among MBC supplementation, gut microbial composition, fecal SCFA profiles, and feed conversion-related parameters, these findings should be interpreted as correlative rather than causal. Future studies incorporating individual feeding systems, nutrient digestibility measurements, microbial functional analyses, targeted metabolomics, strain-specific tracking approaches, intestinal histology, barrier function markers, inflammatory biomarkers, and other host physiological assessments will be necessary to establish causal relationships between microbial ecology, microbial metabolites, and animal performance.

## 5 Conclusion

Dietary supplementation with a microbial bioactive complex (MBC) was associated with improved feed conversion-related parameters in weaned piglets without affecting growth performance or health-related blood parameters. MBC supplementation induced selective modulation of gut microbiota rather than broad changes in microbial diversity, characterized by enrichment of carbohydrate-fermenting and succinate-producing taxa such as *Succinivibrio* and *Succinivibrionaceae* UCG-001. These microbial shifts were accompanied by alterations in gut microbial fermentation profiles, including an increased proportion of propionic acid. Correlation analysis further indicated coordinated relationships among microbial taxa, SCFA composition, and feed conversion-related parameters. Collectively, these findings suggest that MBC supplementation is associated with changes in microbial–metabolic interactions in the gut and may contribute to improved feed conversion performance under practical production conditions. This study supports the potential application of microbial bioactive complexes as functional feed additives in swine production. However, because the present findings are correlative rather than causal, future studies incorporating individual feeding systems, nutrient digestibility measurements, microbial functional analyses, targeted metabolomics, strain-specific tracking approaches, intestinal histology, barrier function markers, inflammatory biomarkers, and other host physiological assessments will be necessary to establish causal relationships between microbial ecology, microbial metabolites, and animal performance.

## Data Availability

The original contributions presented in the study are publicly available. This data can be found here: The raw 16S rRNA gene sequencing data have been deposited in the NCBI Sequence Read Archive (SRA) under accession number PRJNA1244461.
