# Supplementary material for: Microbial bioactive complex supplementation is associated with gut microbial fermentation and feed conversion in weaned piglets
Source: Front Vet Sci. 2026 Jul 9;13:1863429. doi: 10.3389/fvets.2026.1863429 (PMC13391403; doi:10.3389/fvets.2026.1863429)
Supplement: Supplementary file 1 [file Data_sheet_1.docx]

Supplementary Information

Microbial Bioactive Complex Supplementation Is Associated with Gut Microbial Fermentation and Feed Conversion in Weaned Piglets

**Mi Ae Park^1†^, Soyeon Park^1†^, Hee Seop Yu^2^, Da Jung Lim^1^, Seoyun Son^1^, Yong Hee Yoon^2^,** **Dae-Hyuk Kim^1,3^, and Yangseon Kim^1*^**

^1^ Department of Research and Development, Center for Industrialization of Agricultural and Livestock Microorganisms, Jeongeup, 56212, Korea

^2^ Jungnongbio, Jeongeup, 56212, Korea

^3^ Department of Molecular Biology, Department of Bioactive Material Science, Institute for Molecular Biology and Genetics, Jeonbuk National University, Jeonju, 54896, Korea

**
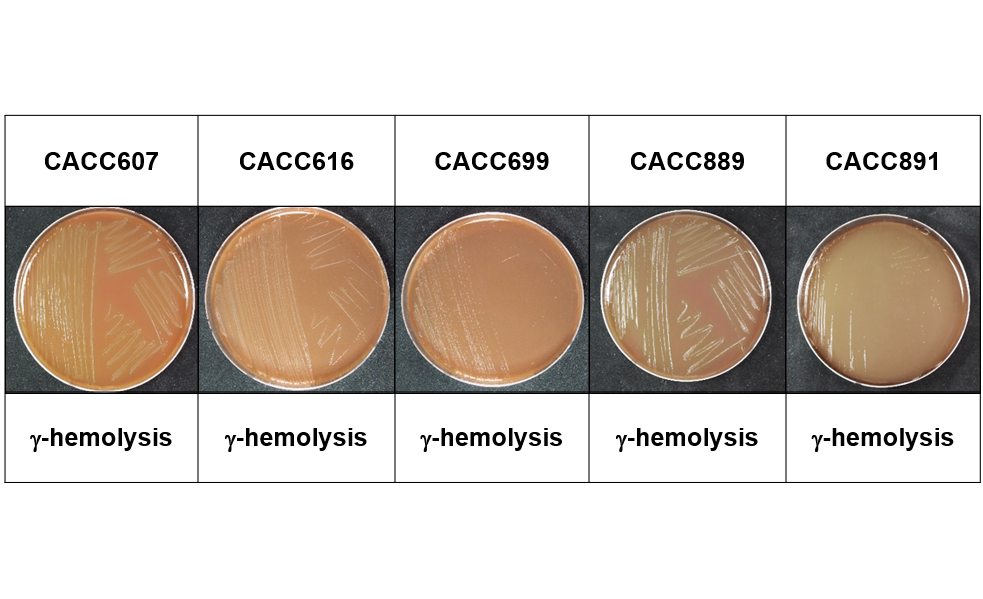
Supplementary Figure S1.** **Hemolytic activity.** Hemolytic activity of *Lactobacillus reuteri* CACC607, *Pediococcus pentosaceus* CACC616, *Saccharomyces cerevisiae* CACC699, *L. dextrinicus* CACC889, and *L. pentosus* CACC891.

**
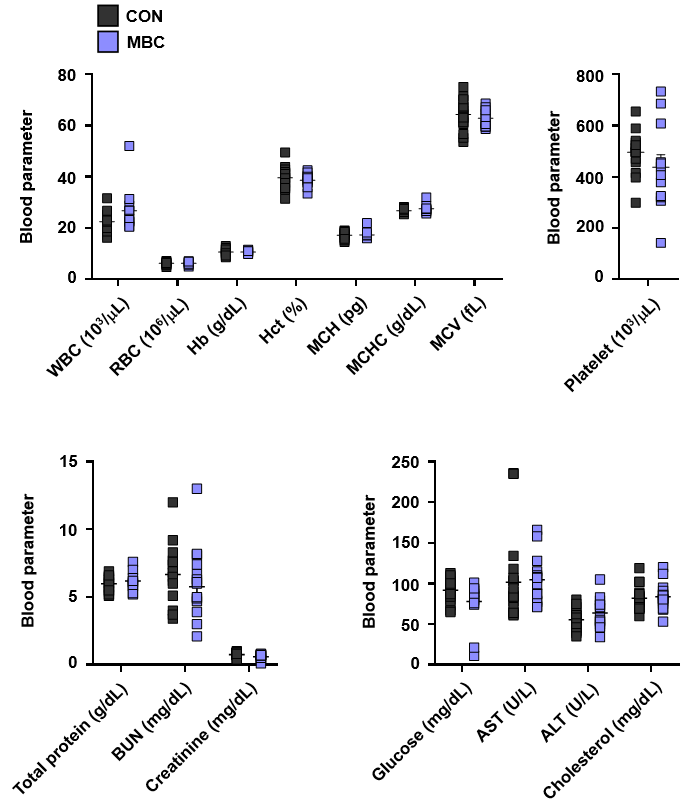
**

**Supplementary Figure S2. Effect of dietary MBC supplementation on blood parameters.** All values are expressed as mean ± SEM (n = 17). WBC, white blood cell; RBC, red blood cell; Hb, hemoglobin; Hct, hematocrit; MCV, mean corpuscular volume; MCH, mean corpuscular hemoglobin; MCHC, mean corpuscular hemoglobin concentration; BUN, blood urea nitrogen; AST, aspartate aminotransferase; ALT, alanine aminotransferase; CON, control diet; MBC, diet supplemented with a microbial bioactive complex produced via solid-state fermentation and containing strains of *Pediococcus*, *Lactobacillus*, and *Saccharomyces*.

**Supplementary Table S1 LEfSe analysis of the gut microbial community in weaned piglets**

| **Group** | **Feature** | **LDA score** | ***P*-values** | ***P*-adj** |
| --- | --- | --- | --- | --- |
| **CON** | *Psychrobacter* | 4.41 | 0.0066 | 0.0066** |
|  | *[Eubacterium] coprostanoligenes group* | 3.26 | 0.0135 | 0.0135* |
|  | *Lachnospira* | 3.17 | 0.0002 | 0.0002*** |
|  | *Ruminococcus 2* | 3.06 | 0.0442 | 0.0442* |
|  | *Shuttleworthia* | 3.02 | 0.0125 | 0.0125* |
|  | *Ruminococcus 1* | 2.90 | 0.0328 | 0.0328* |
|  | *Selenomonas* | 2.86 | 0.0475 | 0.0475* |
|  | *Candidatus Soleaferrea* | 2.69 | 0.0160 | 0.0160* |
|  | *Clostridiales vadinBB60 group* | 2.40 | 0.0260 | 0.0260* |
|  | *Lachnospiraceae AC2044 group* | 2.38 | 0.0002 | 0.0002*** |
|  | *Unclassified WCHB1-41* | 2.19 | 0.0276 | 0.0276* |
|  | *Pseudoramibacter* | 2.13 | 0.0135 | 0.0135* |
|  | *Unclassified Family XIII* | 2.11 | 0.0009 | 0.0009*** |
|  | *Schwartzia* | 2.10 | 0.0045 | 0.0045** |
|  | *Izimaplasmatales* | 2.05 | 0.0132 | 0.0132* |
|  | *Ruminococcaceae UCG-004* | 2.03 | 0.0181 | 0.0181* |
|  | *Intestinimonas* | 2.01 | 0.0328 | 0.0328* |
| **MBC** | *Succinivibrio* | 3.27 | 0.0147 | 0.0147* |
|  | *[Ruminococcus] torques group* | 2.41 | 0.0411 | 0.0411* |
|  | *Glutamicibacter* | 2.33 | 0.0255 | 0.0255* |
|  | *Succinivibrionaceae UCG-001* | 2.23 | 0.0018 | 0.0018** |
|  | *Lachnospiraceae UCG-003* | 2.00 | 0.0435 | 0.0435* |

LEfSe, Linear discriminant analysis effect size (LDA score > 2.0); CON, control diet; MBC, diet supplemented with a microbial bioactive complex produced via solid-state fermentation and containing strains of *Pediococcus*, *Lactobacillus*, and *Saccharomyces*; **P* < 0.05; ***P* < 0.01; ****P* < 0.0001.

**Supplementary Materials and Methods**

**Hemolytic activity**

Hemolytic activity was assessed using blood agar supplemented with 5% (v/v) sheep blood (MBCell, South Korea). Bacterial and yeast cultures grown overnight in their respective media were streaked onto the blood agar plates and incubated for 24 h under their respective growth conditions. Following incubation, hemolytic activity was evaluated by observing red blood cell lysis around the colonies. Strains exhibiting green zones were classified as α-hemolysis, those with clear zones as β-hemolysis, and those without zone formation as γ-hemolysis. Only strains showing γ-hemolysis were considered non-hemolytic and thus regarded as safe (1).

**Blood parameter analysis**

On day 33, blood samples were collected from all pigs for hematological and biochemical analyses. Hematological parameters, including white blood cell count (WBC), red blood cell count (RBC), hemoglobin (Hb), hematocrit (Hct), mean corpuscular volume (MCV), mean corpuscular hemoglobin (MCH), mean corpuscular hemoglobin concentration (MCHC), and platelet count, were analyzed using a BC-6800 Plus analyzer (Mindray, China) according to the manufacturer’s instructions. Biochemical parameters, including total protein, blood urea nitrogen (BUN), creatinine, cortisol, glucose, aspartate aminotransferase (AST), alanine aminotransferase (ALT), and cholesterol, were measured using a Cobas 702 analyzer (Roche Diagnostics, Germany) according to the manufacturer’s instructions.

**References**

1. Mangia NP, Saliba L, Deiana P. Functional and Safety Characterization of Autochthonous Lactobacillus Paracasei FS103 Isolated from Sheep Cheese and Its Survival in Sheep and Cow Fermented Milks during Cold Storage. *Ann. Microbiol.* (2019) 69: 161–70. doi: 10.1007/s13213-018-1416-1
